# Selecting Reaction Pathways of CO_2_ Hydrogenation on Ni(111) by Kinetic Hindrance Associated with the Initial Surface Conditions in the Treatment of H_2_/CO_2_ Mixed Gas, Studied by Ambient-Pressure X‑ray Photoelectron Spectroscopy

**DOI:** 10.1021/acsomega.5c04196

**Published:** 2025-11-21

**Authors:** Yu Murano, Masafumi Horio, Tetsuya Wada, Masashige Miyamoto, Yifu Liu, Yoshinori Kotani, Hiroyuki Yamane, Tetsuya Nakamura, Susumu Yamamoto, Iwao Matsuda

**Affiliations:** † The Institute for Solid State Physics (ISSP), 13143 The University of Tokyo, Kashiwa, Chiba 277-8581, Japan; ‡ Photon Science Innovation Center (PhoSIC), Sendai, Miyagi 980-0845, Japan; § International Center for Synchrotron Radiation Innovation Smart (SRIS), 13101 Tohoku University, Sendai, Miyagi 980-8577, Japan; ∥ Institute of Multidisciplinary Research for Advanced Materials (IMRAM), 13101 Tohoku University, Sendai, Miyagi 980-8577, Japan; ⊥ Institute for Solid State Physics (ISSP), 13143 The University of Tokyo, Kashiwa, Chiba 277-8581, Japan

## Abstract

Two types of surface reaction intermediates were observed in the presence of a H_2_/CO_2_ mixed gas on Ni(111), depending on the dosage order of the gases. The observations were carried out by *in situ* measurements with ambient-pressure X-ray photoelectron spectroscopy. When the CO_2_ gas was introduced at 300 K prior to the H_2_ gas, CO_2_ dissociation occurred. With the opposite order of gas dosage at the same temperature, carboxyl (COOH) formation was observed on the surface. The different reaction pathways originate from initial surface conditions due to the interaction between the first gas and Ni(111). In both cases, the reaction is dominated by COOH formation above 370–420 K, depending on the gas pressure, and is associated with graphitization above 470 K. The different reaction pathways followed according to the dosage order of the gases likely explain the various intermediates observed in previous studies. These results pave a new way to develop CO_2_ hydrogenation catalyst systems with lower energy consumption.

## Graphical abstract

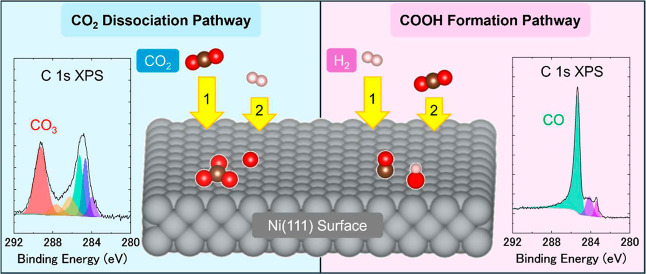

## Introduction

Activation of carbon dioxide (CO_2_) for conversion into fuels or chemical feedstocks for a sustainable society is an important current topic in the chemical and energy industries.^1–3^ CO_2_ can be a source to produce methane using a Ni catalyst. The CO_2_ methanation reaction is known as the Sabatier reaction: CO_2_ + 4 H_2_ → CH_4_ + 2 H_2_O.^4,5^ This reaction competes with the reverse water gas shift (RWGS) reaction (CO_2_ + H_2_ → CO + H_2_O). For over a century, these reaction mechanisms have been a central research issue in catalysis chemistry. Especially, the Sabatier process has recently received attention as a promising renewable energy technique, such as in power-to-gas (P2G) technology.^6^

Mainly, three pathways have been suggested for the initial CO_2_ activation on Ni surfaces in previous studies by X-ray photoelectron spectroscopy (XPS),^7–14^ Fourier transform infrared (FT-IR) spectroscopy,^14–17^ high-resolution electron energy loss spectroscopy,^7,13^ infrared-visible sum frequency generation,^12^ temperature-programmed desorption and reaction spectroscopies,^7,13^ and theoretical calculations.^7,11,13,16,18–23^ One of the pathways is through CO_2_ dissociation into carbon monoxide (CO) and atomic oxygen (O) (CO_2_ → CO + O).^8–10,12,14,16,17,19,20^ The other two pathways proceed through the reaction of CO_2_ and atomic hydrogen (H) on the surface to form carboxyl (COOH)^7,11,13,19,20^ or formate (HCOO).^7,13,15,16^ COOH is formed by O-terminal hydrogenation of CO_2_, while HCOO is formed by C-terminal hydrogenation of CO_2_. When COOH is formed, it is observed as CO and OH (COOH → CO + OH) due to its short lifetime on the surface.^7,11,20,23^ HCOO is stable on Ni(111) and Ni(110) while observed as CO + H (HCOO → HCO → CO + H) on Ni(100).^7,16,23^ HCOO has been observed as a typical intermediate species, especially on Ni nanoparticles on substrates,^15,16^ and the Ni(110) surface.^7,13,19^ On the other hand, almost no formate is experimentally observed on Ni(111)^9–11^ and Ni(100).^10,14,17^

One promising *in situ* experimental method is ambient-pressure X-ray photoelectron spectroscopy (APXPS), in which XPS measurement is carried out on a surface in a gas atmosphere.^24–27^ Previous APXPS experiments were carried out on Ni(111) under 0.2 Torr (0.27 mbar) CO_2_, and the formation of NiO and CO_3_ species at the surface at room temperature (RT) was reported.^9,10^ Subsequent H_2_ dosage and annealing at >423 K resulted in disappearance of these species and, conversely, in appearance of CO and atomic carbon on the surface.^9^ Sequential detection of carbon species (CO and C) has favored a scenario of dissociation of CO_2_ in the Sabatier reaction. In contrast, APXPS research on Ni(111) under a mixture gas of 0.1 mbar CO_2_ and 0.3 mbar H_2_ reported observation of the CO and OH species at 300 K, supporting COOH formation as the reaction mechanism.^11^ These observations have indicated a possible variation of the reaction pathway, depending on the experimental procedure concerning the gas dosage.

The influence of the dosage order of the gases on the reaction pathway of CO_2_ hydrogenation has also been suggested in other APXPS studies on different catalytic surfaces.^28–30^ On Ni(100), the surface is easily oxidized by CO_2_ dissociation when CO_2_ is introduced prior to H_2_, while the surface oxide formation is suppressed by preadsorbed hydrogen when H_2_ is introduced first at RT.^28^ In the case of copper (Cu), the surface was kept metallic when H_2_ was introduced prior to CO_2_, while the surface Cu was oxidized to Cu_2_O and CuO when CO_2_ was introduced prior to H_2_ at 470 K investigated by combining APXPS with near-edge X-ray absorption fine structure (NEXAFS).^29^ The authors have suggested that the oxide should be removed by adding CO since metallic Cu is relevant for CO_2_ activation. Furthermore, in the case of palladium (Pd), CO_2_ dissociated to form CO on the Pd(111) surface when CO_2_ was introduced prior to H_2_, while no CO_2_ dissociation took place when H_2_ was introduced prior to CO_2_ at 300 K.^30^ These results showed that precovered H by H_2_ dissociation prevents CO_2_ activation. More recently, the Ni(111) surface in the presence of CO_2_ and H_2_ has been investigated by combining APXPS with another *in situ* measurement method, polarization modulation infrared photoelectron spectroscopy (PM-IRRAS).^14^ They observed dissociation of CO_2_ into CO and O following CO desorption and H_2_O formation as an RWGS reaction when they introduced CO_2_ prior to H_2_ at about 298 K. When they changed the dosage order of the gases, similar PM-IRRAS spectra were obtained. However, since they focused on the wavenumber region of the C–O stretching of CO under these conditions, the existence of OH or CO_3_ on the surface has remained uncertain.

In the present research, we conducted extensive APXPS experiments on Ni(111) under two types of gas mixture, 2.5 mbar CO_2_/7.5 mbar H_2_ and 0.1 mbar CO_2_/0.3 mbar H_2_ with different dosing orders. Evolutions of the molecules and substrate were monitored by APXPS spectra at the C 1*s*, O 1*s*, and Ni 2*p*_3/2_ core levels. On introducing CO_2_ before H_2_ at 300 K, the surface is dominantly covered with CO, CO_3_, and O (NiO) species, implying the CO_2_ dissociation process. When the dosing order is reversed, CO and OH adsorbates are mainly formed without any Ni oxide on the surface, favoring the mechanism of COOH formation. The two reaction pathways at 300 K are likely ascribed to differences in the surface conditions, with or without Ni oxide, that depend on the initial gas molecule, CO_2_ or H_2_, respectively. When the sample is held at 370 or 420 K, depending on the gas pressure, CO and OH are dominantly generated at the surface by either pathway. These facts consistently explain the results of the previous *insitu* experiments and provide a possible way to regulate the CO_2_ hydrogenation reaction on a surface.

## Experimental Section

A series of APXPS experiments were performed at two synchrotron radiation facilities. One was at beamline BL-13B in the Photon Factory (PF) of the High Energy Acceleration Organization (KEK). The APXPS station at BL-13B is described elsewhere^31,32^ . The other was at beamline BL08U in NanoTerasu. Since the beamline and the APXPS station are newly developed, the details are described in the following subsections.

### Instruments

NanoTerasu BL08U is a high-brilliance soft X-ray beamline with an APPLE–II–type undulator that can generate linearly or circularly polarized soft X-ray light.^33,34^ The beamline optics adopt a collimated plane grating monochromator with four mirrors (M0, M1, M2, M3) and a plane grating (PG)^35^ (see Figure 1a). The electron storage ring is operated at an electron beam energy of 3 GeV and an emittance of 1.14 nm·rad.^36^ A photon energy range of 180–2500 eV can be used with high energy resolving power (>20,000). The X-ray beam is focused on the sample using two postfocusing mirrors (M4 and M5).

**Figure 1:**
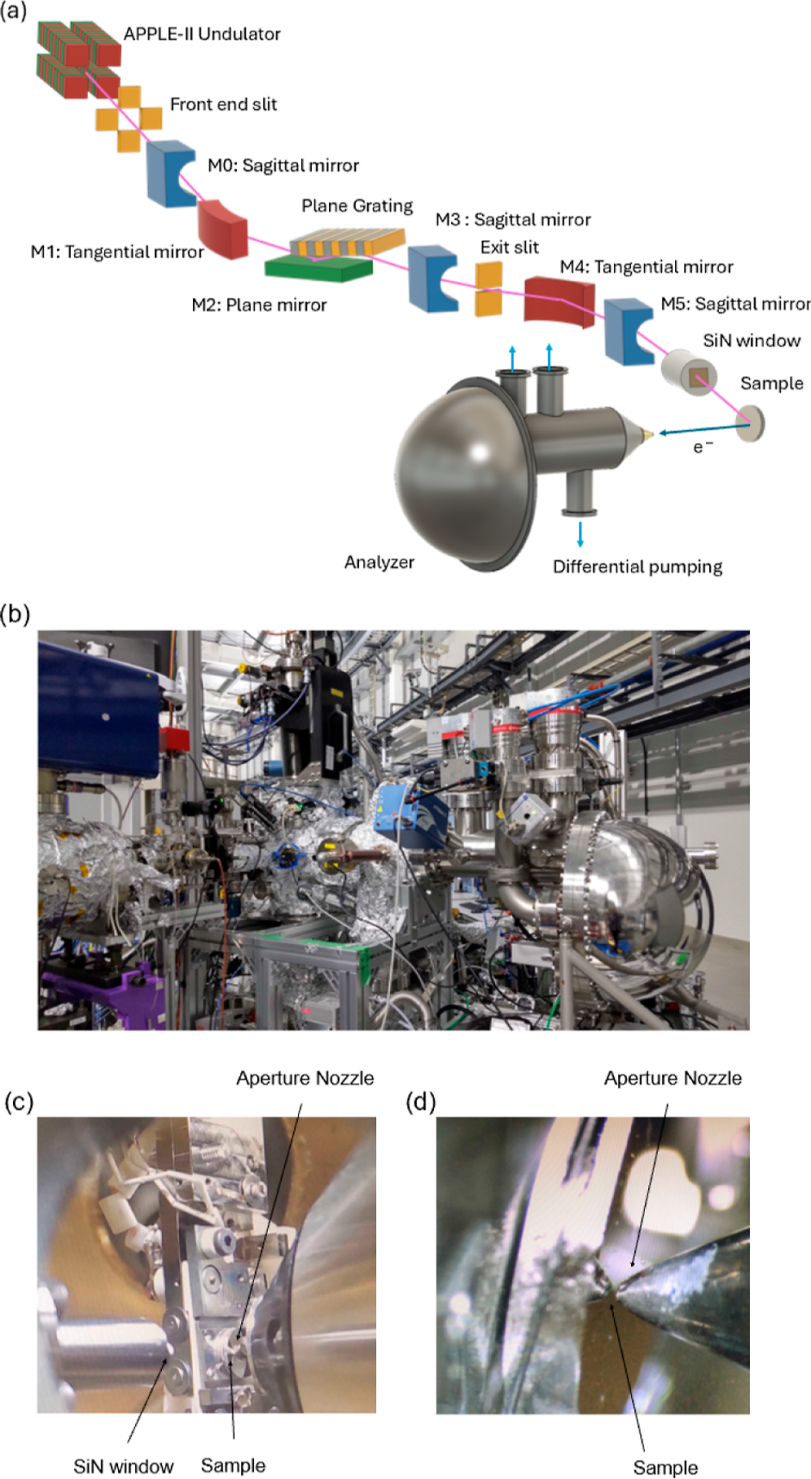
NanoTerasu beamline BL08U and APXPS experimental end station. (a) Schematic drawing. Photographs of (b) end station and (c,d) arrangements in the analysis chamber.

The APXPS system at the beamline end station consists of three interconnected ultrahigh vacuum (UHV) chambers: an analysis chamber, a preparation chamber, and a load-lock chamber (Figure 1b). In the analysis chamber, XPS measurements can be made under both UHV (<6 × 10^–10^ mbar) and ambient (1 bar) pressure conditions.^37^ The pressure is monitored by a combination of a Pirani gauge, an ion gauge, and a capacitance gauge, depending on the pressure range and experimental purpose. The preparation chamber is equipped with an ion source for sample cleaning and electronic optics for low-energy electron diffraction (LEED) observations. The load-lock chamber is used to introduce samples to the analysis chamber.

APXPS measurements were carried out with a differentially pumped electron analyzer (SPECS, PHOIBOS 150 NAP) that consists of a differentially pumped electrostatic prelens and a three-stage differentially pumped hemispherical analyzer. Electrons are detected by a 1D delay-line detector. A backfilling APXPS experiment was adopted for the present system. To achieve the APXPS measurements,^37^ we appropriately set the minimum distance between the aperture nozzle and the sample, accordingly restricting the X-ray beam in the grazing incidence arrangement (Figure 1c). In addition, a vacuum tube, terminated by a SiN window (200 nm thickness), was installed inside the analysis chamber to minimize the length of the X-ray beam path under ambient pressure. The incident angle of the X-ray beam and emission angle of the photoelectrons were set to 80° and 10°, respectively, with respect to the surface normal.

### Sample Preparation

A surface of a Ni(111) single crystal (MaTecK Co., Ltd.) with ϕ 10 mm and ^*t*^1.5 mm was cleaned by repeated cycles of Ar^+^ ion sputtering (15 min, 1 keV, 2–3 μA) and annealing at 780 °C (10 min). Cleanliness of the sample surface was checked by measuring the X-ray photoelectron spectroscopy (XPS) survey spectrum, and the orderliness of the surface structure was confirmed by the low-energy electron diffraction (LEED) pattern under ultrahigh vacuum (UHV) conditions.

The reactant gases were H_2_ (Taiyo Nippon Sanso Corp., 99.99999% purity) and CO_2_ (Taiyo Nippon Sanso Corp., 99.995% purity).

### APXPS Measurements

Ambient-pressure XPS (APXPS) measurements were made at photon energies of *h*ν = 490 eV for the C 1*s* level, *h*ν = 730 eV for the O 1s level, and *h*ν = 1050 eV for the Ni 2*p*_3/2_ level. The photon energies were selected so that the kinetic energies (*E*_kin_) of the photoelectrons give similar inelastic mean free path (IMFP) for each region (C 1*s*, O 1*s*, and Ni 2*p*). At *E*_kin_ = 200 eV, adopted in the present experiment, IMFP was evaluated as 0.5 nm for Ni metal, as calculated by QUASES-IMFP-TPP2M software.^38^ This showed that the probing depth of the APXPS data was sufficiently surface-sensitive. To avoid possible beam-induced effects, the X-ray beam was only irradiated on the sample surface during the APXPS measurements. For the APXPS at NanoTerasu BL08U, the hole size of the aperture nozzle of the analyzer was 300 μ m and so was the distance between the sample and the aperture nozzle (Figure 1d).

Binding energies (BEs) refer to the Fermi level (*E*_F_) of the Ni(111) substrate. Spectral analysis was carried out using Casa XPS software. A Shirley background was used for all core-level spectra (C 1*s*, O 1*s*, and Ni 2*p*_3/2_). All the curve fitting was undertaken using a Gaussian (70%)–Lorentzian (30%) line shape, except for asymmetric CO peaks, in accordance with previous studies.^10,39,40^ The binding energy, width, and asymmetry were fixed for each peak throughout the analyses. The atomic ratio of carbon with respect to nickel (C/Ni) was calculated based on the peak intensity ratio of C 1*s* with respect to Ni 2*p*_3/2_ by accounting for each cross-section and IMFP.

Quadrupole mass spectroscopy (QMS) was also measured during the APXPS measurements, while neither CH_4_ nor CO production was detected under the experimental conditions. This is probably due to the small surface area of low-index single crystals and the long distance between the sample position and the QMS detector, which is equipped at the third stage of the differential pumping.

## Results and Discussion

### Case When CO_2_ Gas Is Introduced First

Figure 2 shows a series of (a) C 1*s*, (b) O 1*s*, and (c) Ni 2*p*_3/2_ spectra taken at KEK-PF BL-13B for the various processes: in the presence of 0.1 mbar of CO_2_, after the addition of 0.3 mbar of H_2_, during sample annealing, and after evacuation of the CO_2_/H_2_ mixed gases. Spectral features of the C 1*s* and O 1*s* spectra were deconvoluted to the fitted peaks with binding energies (BEs) and the full width at half-maximum (fwhm) of the C 1*s* and O 1*s* components summarized in Table 1. The C/Ni atomic ratio as a function of temperature is shown in Figure 4a.

**Figure 2:**
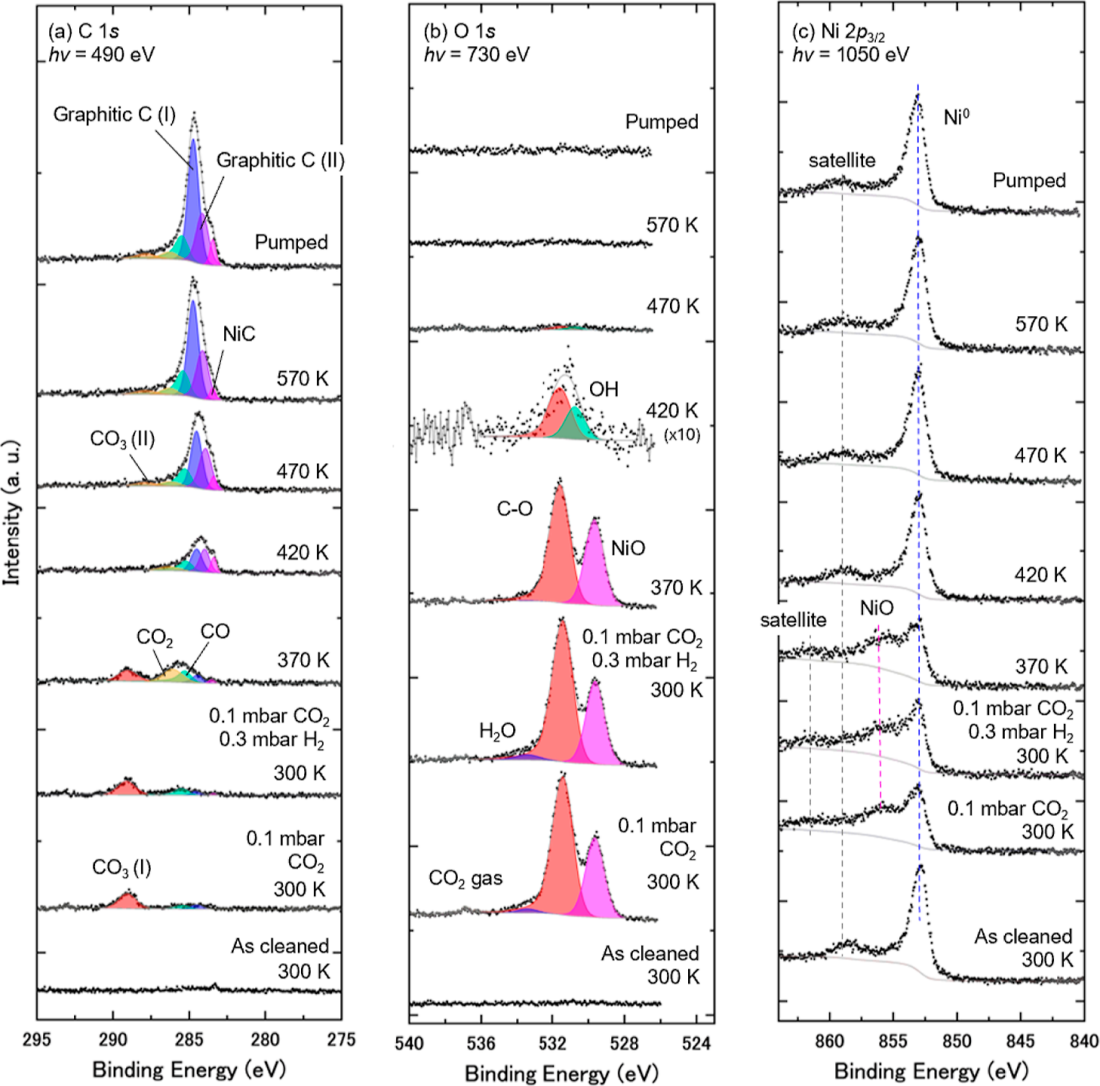
A series of APXPS of Ni(111) in the presence of CO_2_ and H_2_ gases, taken at core levels of (a) C 1*s*, (b) O 1*s*, and (c) Ni 2*p*_3/2_. The surface was exposed to 0.1 mbar CO_2_ and then mixed with 0.3 mbar H_2_ at 300 K. The sample was subsequently annealed at 370, 420, 470, and 570 K, followed by gas evacuation to the UHV condition at 570 K. Curve-fitting results are shown in (a,b). The Ni 2*p*_3/2_ core-level spectra are normalized by the peak area of Ni 2*p*_3/2_ for comparison. The C 1*s* and O 1*s* spectra are normalized with their background intensities.

**Table 1:** Summary of Binding Energies (BEs) and Full Width at Half-Maximum (FWHM) of Components in C 1*s* and O 1*s* Spectra

	C 1*s*		O 1*s*		refs
	BE (eV)	fwhm (eV)	BE (eV)	fwhm (eV)	
CO_3_ (I)	289.2 ± 0.1	1.3 ± 0.1	531.4 ± 0.1	1.3 ± 0.1	^8–12,48^
CO_3_ (II)	287.7 ± 0.1	1.3 ± 0.1	531.4 ± 0.1	1.3 ± 0.1	^7,13^
CO_2_	286.2 ± 0.1	1.3 ± 0.1	531.4 ± 0.1	1.3 ± 0.1	^7,13^
CO	285.4 ± 0.1	0.8 ± 0.1	531.4 ± 0.1	1.3 ± 0.1	^8–12,39^
Graphitic C (I)	284.6 ± 0.1	0.7 ± 0.1			^7,8,10–12^
Graphitic C (II)	284.0 ± 0.1	0.8 ± 0.1			^7,8,10–12^
NiC	283.4 ± 0.1	0.4 ± 0.1			^8,45,46^
H_2_O			533.3 ± 0.1	1.4 ± 0.1	^7,10,11,47^
OH			530.9 ± 0.1	1.1 ± 0.1	^10,47,49,50^
NiO			529.4 ± 0.1	1.0 ± 0.1	^9,49–51^

**Figure 3:**
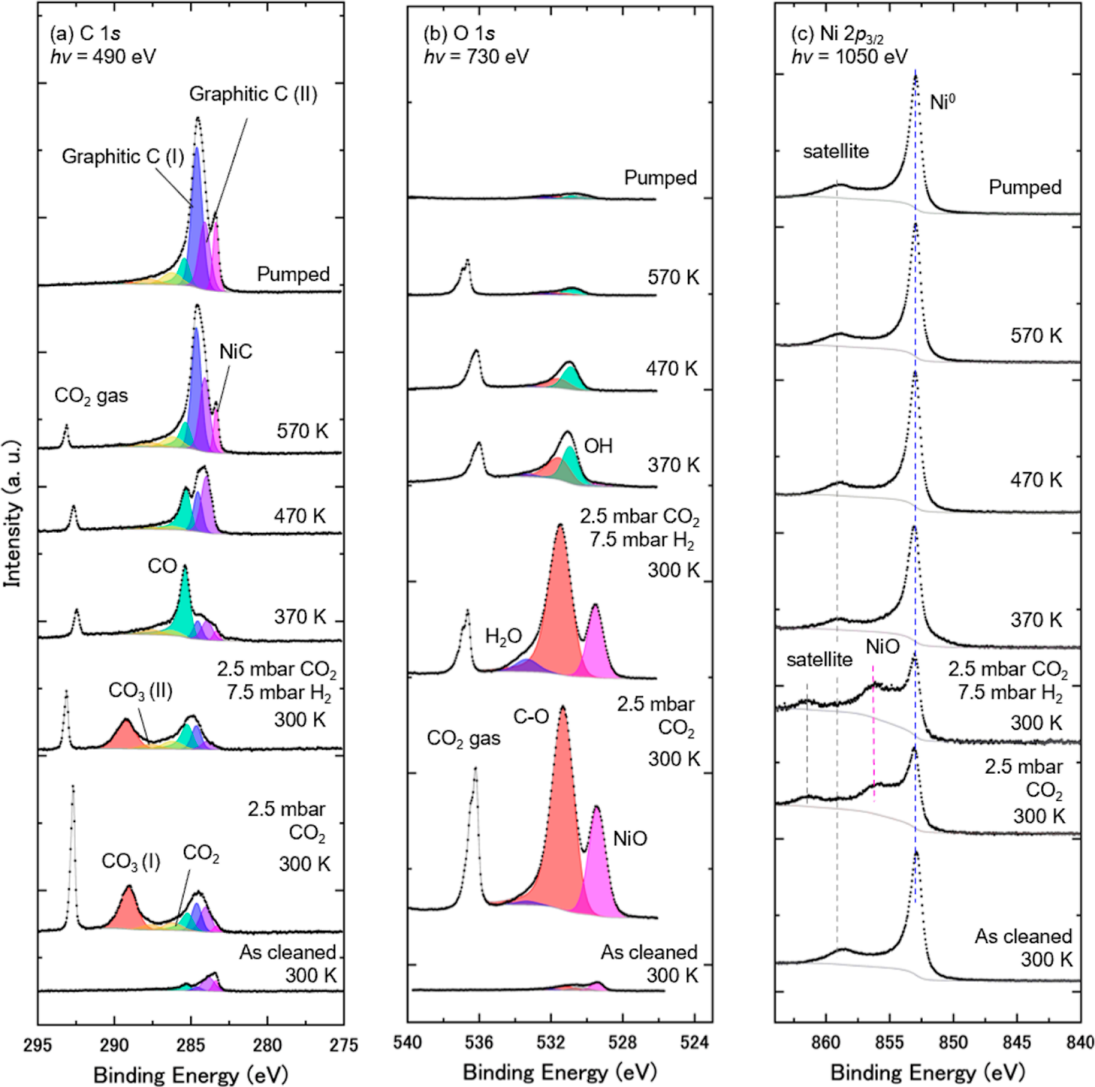
A series of APXPS of Ni(111) in the presence of CO_2_ and H_2_ gases, taken at core levels of (a) C 1*s*, (b) O 1s, and (c) Ni 2*p*_3/2_. The surface was exposed to 2.5 mbar CO_2_ and then mixed with 7.5 mbar H_2_ at 300 K. The sample was subsequently annealed at 370, 470, and 570 K, followed by gas evacuation to the UHV condition at 570 K. Curve-fitting results are shown in (a,b). The Ni 2*p*_3/2_ core-level spectra are normalized by the peak area of Ni 2*p*_3/2_ for comparison. The C 1s and O 1s spectra are normalized with their background intensities.

**Figure 4:**
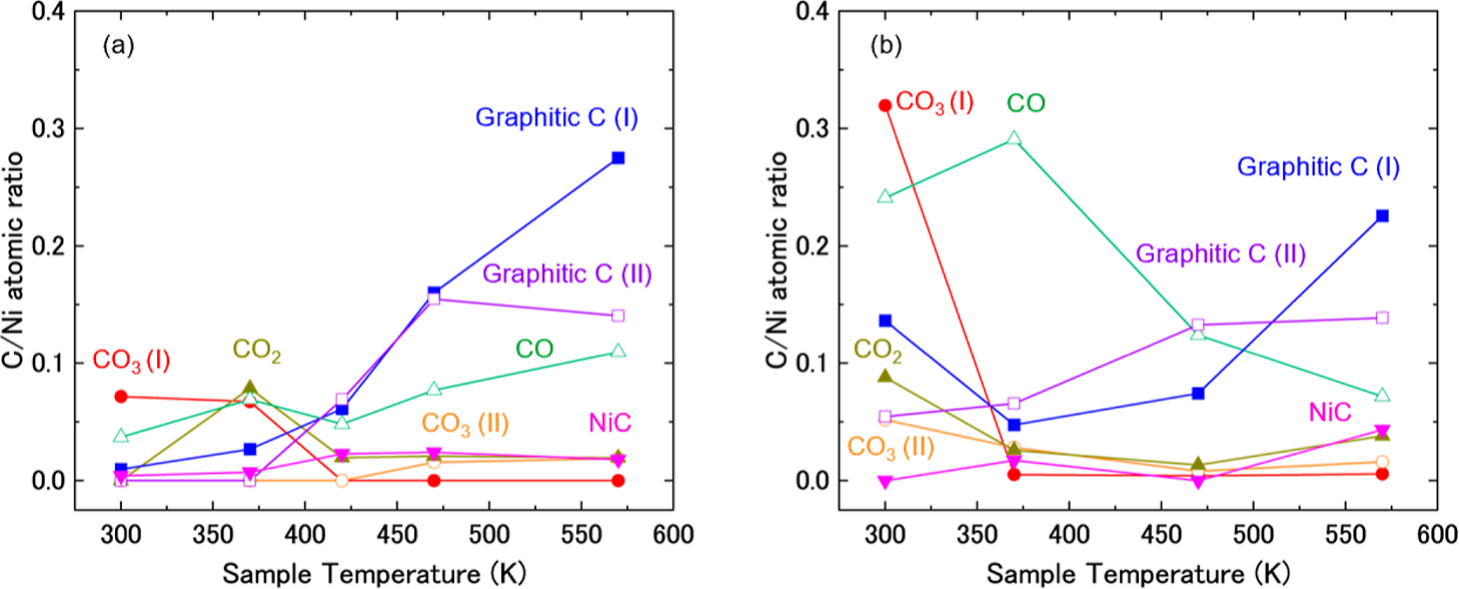
C/Ni atomic ratio calculated based on APXPS spectra taken at various temperatures on Ni(111) in the presence of (a) 0.1 mbar CO_2_ and 0.3 mbar H_2_ and (b) 2.5 mbar CO_2_ and 7.5 mbar H_2_, with CO_2_ gas initially dosed into the analysis chamber.

Through a series of experimental procedures, seven components were obtained in C 1*s* spectra by the curve-fit analysis, as given in Figure 2a and Table 1. The components at BEs of 289.2 and 285.4 eV are ascribed to carbonate (CO_3_(I)) and CO, respectively. These assignments are in agreement with previous APXPS studies on Ni(111).^8–12^ These BE values indicate that the CO molecules adsorb at hollow sites, rather than top sites.^9,39^ Peaks located between those of CO_3_(I) and CO can naturally be ascribed to CO_2_ or a related structure; thus, the component at 286.2 eV likely corresponds to the adsorbed CO_2_ species.^41,42^ This BE position is the same as that of the CO_2_ adsorbate on Ni(110),^7,13^ which supports this assignment. The component at 287.7 eV is located close to the binding energy of CO_3_ (I) and shows a similar behavior with temperature. This species is related to CO_3_, and the lower BE value implies that it has a chemically different environment. It is thus reasonable to consider this component as CO_3_(II) in a different adsorption configuration. In contrast, an XPS study^7,13^ on Ni(110) reported the core-level peak of the bidentate formate (HCOO) at BE = 287.7 eV. These assignments share the same BE positions, so there may be spectral overlaps of the CO_3_(II) and HCOO species after the H_2_ dosage. Based on previous studies, the peaks at 284.6 and 284.0 eV are ascribed to graphitic carbon^7,10–12^ at the different adsorption sites,^8^ while the peaks observed at 300 K can also contain the components of contaminant hydrocarbons.^9,43^ Since pure *sp* ^2^-hybridized carbons show satellites above 290 eV,^44^ which were not observed in this study, the carbon species are not pure graphite but a mixture of *sp* ^2^- and *sp* ^3^-hybridized carbons. The peak at 283.4 eV is attributed to the surface carbon atoms that take a similar chemical environment as in nickel carbide (NiC),^8,45,46^ while the negligible peak at 300 K can also contain atomic carbon.^9,11^

Four components were identified in the O 1*s* spectra, as given in Figure 2b and Table 1. The peak at 533.3 eV can energetically be assigned to adsorbed water molecules (H_2_O), as previously reported.^7,10,11,47^ Chemical species at 530.9 and 531.4 eV are ascribed to oxygen atoms that bond with hydrogen atoms (−OH) and carbon atoms (CO_3_, CO_2_, and CO), respectively.^7,9,10,13,47–50^ The component at 529.4 eV originates from nickel oxide (NiO) at the surface.^9,49–51^ The Ni 2*p*_3/2_ spectra have contained features of Ni metal (Ni^0^) around 853 eV and Ni oxides (NiO) around 855 eV, along with their satellites,^9,11,49,50,52,53^ as shown in Figure 2c. The intensity around 530.1 eV observed in the O 1*s* region after pumping could be assigned to Ni­(OH)_2_ based on the binding energy, according to previous studies.^49,50^

Under 0.1 mbar of CO_2_ at 300 K, CO_3_ and NiO are the dominant adsorbed species. This indicates that CO_2_ dissociation (CO_2_ → CO + O) proceeds and that the adatoms react with Ni to form NiO (Ni + O → NiO) and with the other CO_2_ molecules to produce CO_3_ (CO_2_ + O → CO_3_). Thus, it is inferred that CO_3_ is the most dominant C–O species at the surface and the O 1*s* peak at 531.4 eV is governed by CO_3_ (Table 1). On adding 0.3 mbar of H_2_, the spectral features of the CO species and H_2_O are enhanced. These data likely indicate that H_2_ promotes the dissociation of CO_2_ and initiates H_2_O formation at the surface. Appearance of a negligible peak of H_2_O after the CO_2_ dosage, shown in Figure 2b, seemingly resulted from adsorption of residual H_2_ gas in the analysis chamber, following the same scenario of H_2_O formation.^9,47,54,55^

Figure 2 shows significant spectral changes when the sample was annealed, and the C 1*s* spectra appear to provide detailed information at the surface. To capture the spectral evolution of the carbon species, Figure 4a shows a plot of the carbon/nickel (C/Ni) atomic ratio as a function of temperature. The CO_3_ and NiO species are apparently reduced at 420 K, and the CO and OH species appear on the surface. This tendency is consistent with the previous APXPS study under typical low-pressure conditions below 0.5 mbar, in which CO_3_ and NiO disappear at 425 to 430 K.^9^ The carboxyl (COOH) species can be generated by CO_2_ that reacts with H adatoms (CO_2_ + H → COOH). COOH easily dissociates into CO and OH at this temperature,^11,19,22,56^ so the observation of CO and OH species provides evidence of COOH formation at the surface. Above 470 K, these species decrease, and those of graphitic carbon increase. H atoms at the hollow site are gradually replaced by stable C atoms, forming graphitic carbons at higher temperatures.^45,57–59^ This feature corresponds to the accumulation of carbon, which inactivates the Sabatier reaction on Ni catalysts.^40,45,46,59,60^ The C 1*s* spectra at 570 K after pumping of the mixed CO_2_/H_2_ gas are identical to those taken *in situ* at 570 K, and graphitic carbon remained stable at the surface. To confirm that the carbon species evolution at high temperatures comes from the reaction between the reactant gases and the Ni(111) surface, we measured XPS spectra at annealing temperatures without gases (Figure S1). The results show that the carbon species segregated during heating is observed as NiC, while graphitic carbons generated by heating are smaller than those under the reactant gases (Figure S2). This indicates that the large amounts of graphitic carbon evolving above 470 K in Figures 2, 3, 5, and 6 are from the atomic carbons formed by the interaction between the sample surface and the reactant gases.

**Figure 5:**
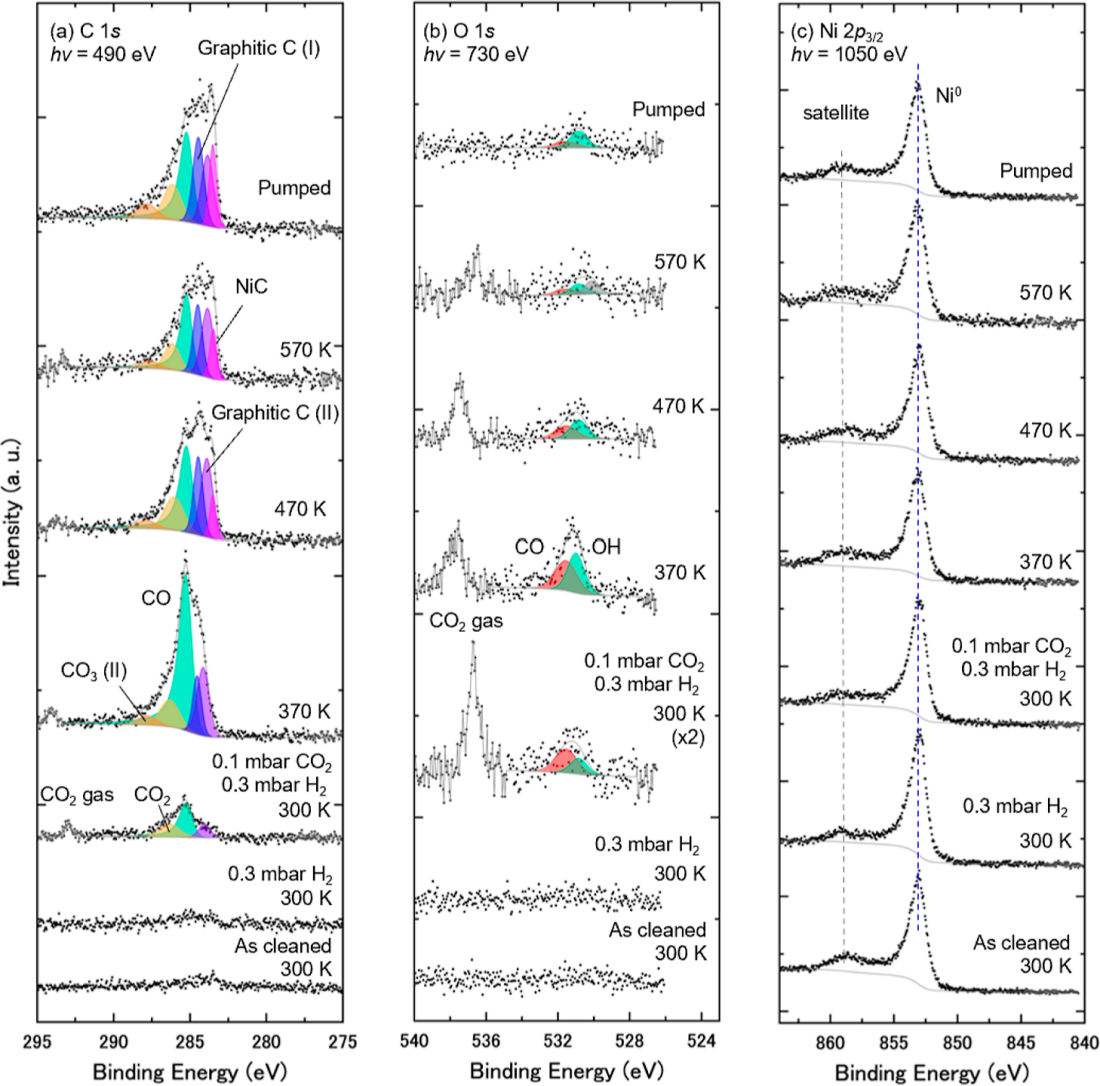
A series of APXPS of Ni(111) in the presence of H_2_ and CO_2_ gases, taken at core levels of (a) C 1*s*, (b) O 1*s*, and (c) Ni 2*p*_3/2_. The surface was exposed to 0.3 mbar H_2_ and then mixed with 0.1 mbar CO_2_ at 300 K. The sample was subsequently annealed at 370, 470, and 570 K, followed by gas evacuation to the UHV condition at 570 K. Curve-fitting results are shown in (a,b). The Ni 2*p*_3/2_ core-level spectra are normalized by the peak area of Ni 2*p*_3/2_ for comparison. The C 1*s* and O 1*s* spectra are normalized with their background intensities.

**Figure 6:**
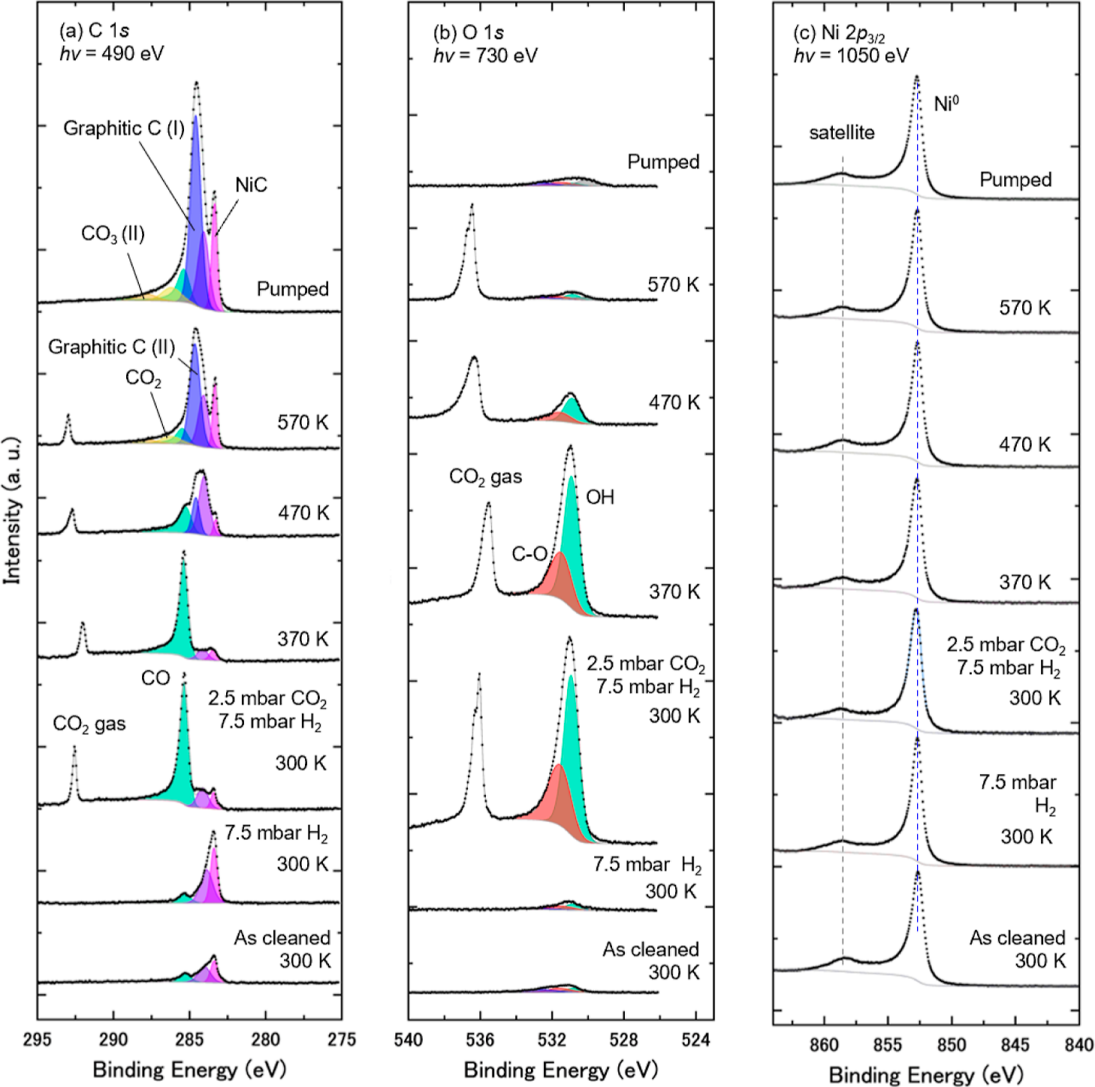
A series of APXPS of Ni(111) in the presence of H_2_ and CO_2_ gases, taken at core levels of (a) C 1*s*, (b) O 1*s*, and (c) Ni 2*p*_3/2_. The surface was exposed to 7.5 mbar H_2_ and then mixed with 2.5 mbar CO_2_ at 300 K. The sample was subsequently annealed at 370, 470, and 570 K, followed by gas evacuation to the UHV condition at 570 K. Curve-fitting results are shown in (a,b). The Ni 2*p*_3/2_ core-level spectra are normalized by the peak area of Ni 2*p*_3/2_ for comparison. The C 1*s* and O 1*s* spectra are normalized with their background intensities.

Furthermore, we conducted another APXPS experiment at NanoTerasu BL08U under much higher pressures of reactant gases. Figure 3 shows a series of (a) C 1s, (b) O 1s, and (c) Ni 2*p*_3/2_ spectra taken for the various processes: in the presence of 2.5 mbar CO_2_, after the addition of 7.5 mbar H_2_, during sample annealing, and after evacuation of the CO_2_/H_2_ mixed gases. It is of note that peaks for the CO_2_ gas are located above 292 and 535 eV in the C 1*s* and O 1*s* spectra, respectively. The spectral assignments and curve fittings follow those of Figure 2. Figure 4b presents changes in the C/Ni atomic ratio as a function of temperature.

Under 2.5 mbar CO_2_ at 300 K and also after adding 7.5 mbar H_2_, CO_3_ and NiO are the dominant adsorbed species, which is consistent with the results under low-pressure conditions in Figure 2. This is confirmed by considering the ratio of peak areas of the O 1s at 531.4 eV and C 1*s* components at 289.2 eV. After normalization of peak area intensity to the CO_2_ gas peaks, the ratio is given as O/C = 3.3, which is consistent with the chemical stoichiometry of CO_3_.

CO_3_ and NiO disappear at 370 K, which is a lower temperature compared to the low-pressure condition (420 K). This is probably due to the high pressure of H_2_ gas, which dissociates on the surface and reacts with those species. After the disappearance of CO_3_ and NiO at 370 K, CO and OH become dominant, indicating the COOH formation process on the surface, which is consistent with the result under the low-pressure condition (Figure 2). Above 470 K, carbon species such as graphitic carbons accumulate on the surface, which is also consistent with the result of Figure 2. Those similarities in the results of Figures 2 and 3 indicate that the contaminants on the “as cleaned” surface in Figure 2, whose C/Ni atomic ratios are 0.03 for NiC, 0.009 for graphitic C (I), 0.05 for graphitic C (II), and 0.02 for CO, do not affect the reaction pathways.

Comparing Figure 4a with [Fig fig4]b, the C/Ni atomic ratio of CO_3_(I) and CO at low temperatures is higher under high-pressure conditions (Figure 4b) compared to the low-pressure condition (Figure 4a). This is seemingly due to the high pressure of reactants (CO_2_/H_2_) to form those intermediate species.

### Case When H_2_ Gas Is Introduced First

Figure 5 shows a series of (a) C 1*s*, (b) O 1*s*, and (c) Ni 2*p*_3/2_ spectra taken at KEK-PF BL-13B for the various processes: in the presence of 0.3 mbar H_2_, after the addition of 0.1 mbar CO_2_, during sample annealing, and after evacuation of the mixed gases. The spectral assignments and curve fittings follow those of Figure 2. BEs and fwhm data for the C 1*s* and O 1*s* components are summarized in Table 1. Figure 7a presents the change in the C/Ni atomic ratio with temperature.

**Figure 7:**
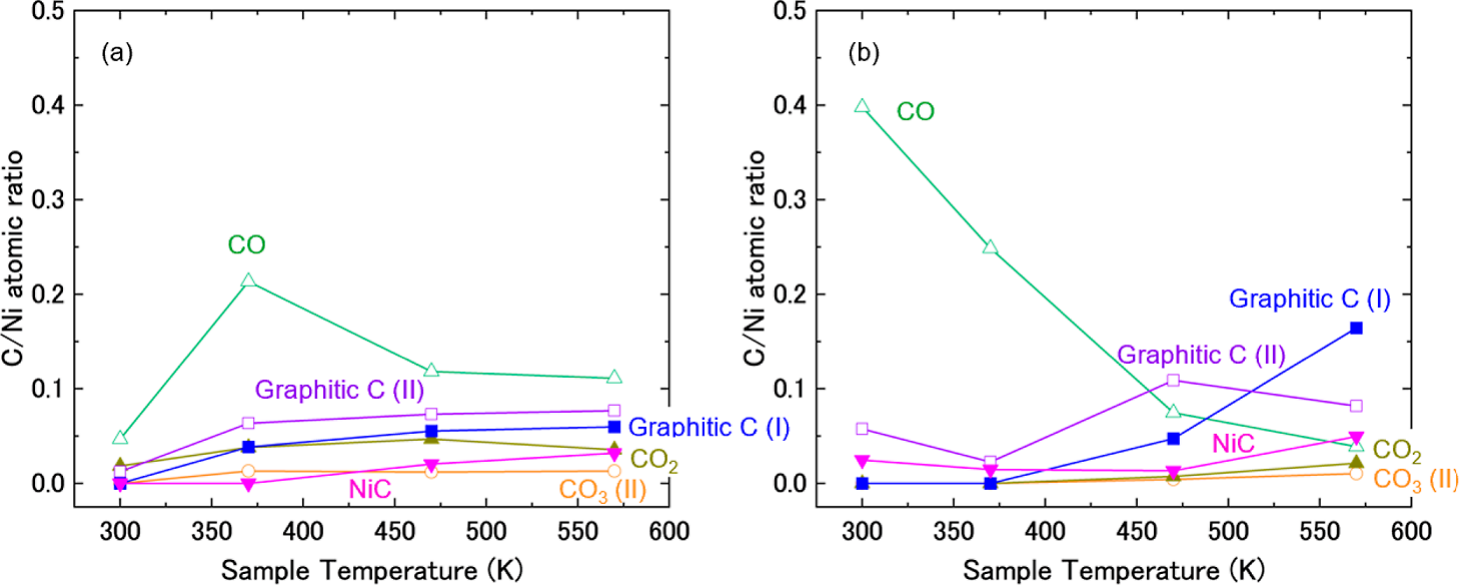
C/Ni atomic ratio calculated based on APXPS spectra taken at various temperatures on Ni(111) in the presence of (a) 0.1 mbar CO_2_ and 0.3 mbar H_2_ and (b) 2.5 mbar CO_2_ and 7.5 mbar H_2_, with H_2_ gas initially dosed into the analysis chamber.

Throughout all the processes, no NiO signal was detected in the Ni 2*p*_3/2_ spectra. This is apparently due to the initial introduction of 0.3 mbar H_2_ at 300 K. Under 0.3 mbar H_2_ at 300 K, no carbon or oxygen species are observed, indicating that there are no contaminants due to the H_2_ dosing. On adding 0.1 mbar CO_2_ gas, CO and OH are observed, indicating the COOH formation process at the surface, while their amounts are relatively small. At 370 K, CO and OH become dominant adsorbed species, indicating COOH formation is more active on the surface at 370 K than at 300 K under this low-pressure condition. Above 470 K, these species apparently reduce and the graphitic carbon components increase, resulting from the replacement of H atoms adsorbed at hollow sites by stable C atoms. The graphitic carbon and NiC species remained after pumping.

Furthermore, we conducted another APXPS experiment at the NanoTerasu BL08U under much higher pressures of reactant gases. Figure 6 shows a series of (a) C 1*s*, (b) O 1*s*, and (c) Ni 2*p*_3/2_ spectra taken for the various processes: in the presence of 7.5 mbar of H_2_, after the addition of 2.5 mbar of CO_2_, during sample annealing, and after evacuation of the CO_2_/H_2_ mixed gases. The spectral assignments and curve fittings follow those of Figure 2. The C/Ni atomic ratio as a function of the temperature is shown in Figure 7b.

In the C 1*s* spectrum, a certain amount of carbon species appeared after the H_2_ dosage, which is likely due to adsorption of carbon contaminants that desorb from the analysis chamber wall in exchange for the hydrogen adsorption. On adding 2.5 mbar of CO_2_ at 300 K, the dominant species observed were CO and OH, indicating the COOH formation process at the surface, which is consistent with the results under low-pressure conditions (Figure 5). This situation was preserved at a sample temperature of 370 K. The fact that the amounts of CO and OH are already large at 300 K (Figure 7b) compared to low-pressure conditions (Figure 7a) is related to the tendency in Figure 2 that CO_3_ and NiO disappear at higher temperature under the low-pressure condition (420 K) compared to the high-pressure condition (370 K, Figure 3). High-pressure reactant gases make it possible for the reaction to proceed at a lower temperature compared to under low-pressure conditions. Above 470 K, carbon species including graphitic carbons and NiC accumulate on the surface, which is also consistent with the result under the low-pressure condition (Figure 5a). Those similarities in the results of Figures 5 and 6 indicate that the contaminants on the “as cleaned” surface in Figure 5, whose C/Ni atomic ratios are 0.02 for NiC, 0.02 for graphitic C (II), and 0.01 for CO, do not affect the reaction pathways.

Comparing Figure 7a with [Fig fig7]b, the amount of CO at low temperatures is larger under the high-pressure condition (Figure 7b) compared to the low-pressure condition (Figure 7a). This is probably due to the high-pressure reactants (CO_2_/H_2_) forming CO as an intermediate. On the other hand, CO remains on the surface above 470 K in Figure 7a, while it decreases rapidly in Figure 7b. This tendency can also be explained that the carbon species including CO can be easily consumed at a higher pressure of H_2_, especially at high temperatures.

Results for the initial processes are sharply in contrast to the case in which CO_2_ gas was introduced first, followed by H_2_ gas. There were no pathways for CO_2_ dissociation at the surface, and the phenomenon is directly related to the initial dosage of H_2_ gas. At 300 K, dissociative adsorption of the H_2_ molecule is held on Ni(111) and the surface is covered with atomic hydrogen.^11,22,57^ It is likely that such hydrogen adatoms prevent CO_2_ dissociation. This is consistent with previous studies that reported both atomic hydrogen and adsorbed species formed by CO_2_ dissociation (CO and atomic oxygen) can stably be located at the hollow site on Ni(111).^9,39,57,58,61–63^ Thus, dissociation of CO_2_ is unlikely to occur when the hollow sites are already occupied by atomic hydrogen. On the other hand, at high temperatures, the two cases are similar: the CO and OH species are dominant at the surface at 370 or 420 K, depending on the gas pressure; above 470 K, these components reduce and graphitic carbon becomes dominant, despite the CO remaining on the surface under low-pressure condition when H_2_ is introduced first (Figure 5). To isolate the effects of gas-dosing order at high temperatures before reaching thermal equilibrium, measurements with enough time resolution will be needed.^64^ It is worth mentioning that an O atom in the NiO phase is at the hollow site of the Ni(111) surface.^61–63^ After the disappearance of NiO due to the reaction with H_2_ at 370 or 420 K, depending on the gas pressure, H atoms can be adsorbed on the hollow sites, leading to COOH formation.^9,47^ These APXPS experimental results show distinctive surface reactions depending on the dosage order of the CO_2_/H_2_ gas. This comprehensive data set, including spectra of the Ni substrate, directly indicates that the different reaction pathways originate from the initial surface conditions due to interaction between the first gas and Ni(111).

### Beam-Induced and Pressure Effects

The high brilliance X-ray beam, generated at the synchrotron radiation facility, has allowed us to conduct *in situ* observation of the sample surface by APXPS, while the beam itself may also induce possible chemical phenomena that should be examined carefully. Based on the previous research of the beam-induced effect,^65,66^ we examined the issue by reducing the incident photon flux by more than 1 order of magnitude and probed a fresh area on the surface that has not been exposed to the X-ray beam. Under such an experimental condition, a series of (a) C 1*s*, (b) O 1*s*, and (c) Ni 2*p*_3/2_ spectra were recorded at NanoTerasu BL08U for a Ni(111) surface under 0.1 mbar CO_2_ at 300 K and also with the subsequent addition of 0.3 mbar H_2_, as shown in Figure 8. The spectral assignments and curve fittings follow those in Figure 2. It is of note that the pressures of CO_2_ and H_2_ were also reduced to match the previous studies^9–11,14^ for examining a possible influence of partial pressures of the reactant gases.^67^ Figure 8 shows that all the APXPS spectra, taken under the conditions of reduced flux and decreased gas pressure, are similar to those in Figure 3. This fact indicates that CO_2_ dissociation, as well as the following NiO and CO_3_ formation, are the essential catalytic phenomena without any contribution of the beam-induced or gas pressure effect. Namely, atomic oxygen is produced in the CO_2_ dissociation process (CO_2_ → CO + O), followed by the formation of NiO and CO_3_ species. It is worth mentioning that the atomic oxygen (O), formed by the CO_2_ dissociation, can be removed by the residual H_2_ gas,^30^ and the CO_3_ species may not be observable on the Ni(111) surface in the presence of 1 mbar CO_2_ at room temperature.^14,17^ Since the beam-induced effect was found not to contribute to the CO_3_ formation, the role of the residual hydrogen gas is seemingly important to understand why CO_3_ was not observed in some cases of the previous experiments.

**Figure 8:**
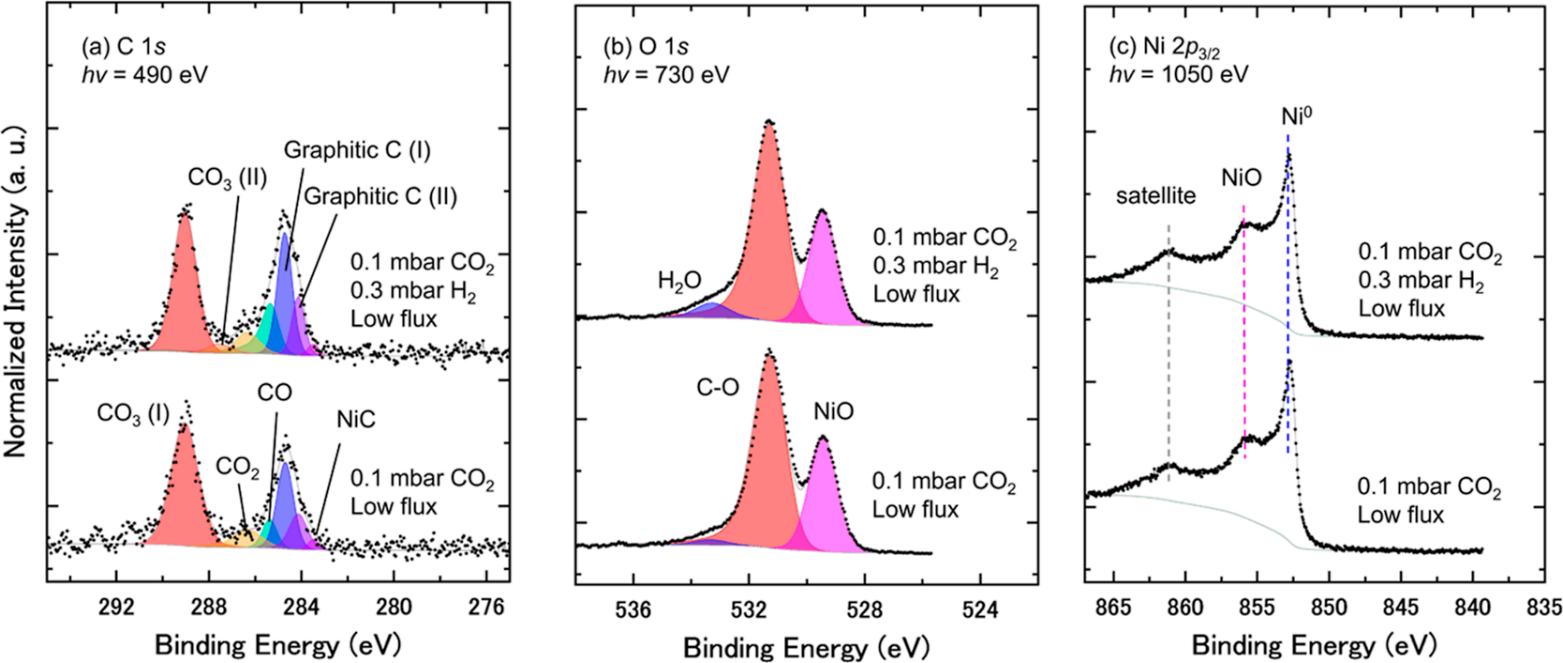
A series of (a) C 1*s*, (b) O 1*s*, and (c) Ni 2*p*_3/2_ spectra taken under 0.1 mbar CO_2_ and after adding 0.3 mbar H_2_, measured with low (reduced) photon flux at each fresh spot. Compared to the measurements for the spectra in Figure 3, the photon flux was reduced to (a) 1/80, (b) 1/60, and (c) 1/12. All the APXPS spectra were taken at 300 K. Curve-fitting results are shown in (a,b). The photon energy for C 1*s*, O 1*s*, and Ni 2*p*_3/2_ core-levels is 490, 730, and 1050 eV, respectively. C 1*s*, O 1*s*, and Ni 2*p*_3/2_ spectra are normalized by the peak area of Ni 2*p*_3/2_ for comparison.

### Initial Chemical States in the Presence of CO_2_ and H_2_ on Ni(111)

The CO_2_ hydrogenation reaction has been historically investigated and has recently become a major focus of attention due to its importance in achieving a sustainable society. Improvements in its industrial applications are continuously demanded, and its mechanisms have been research topics in basic chemistry of catalysis. These APXPS observations have revealed that the reaction process on a Ni(111) surface depends on the dosage order of the CO_2_/H_2_ mixed gas. On introducing CO_2_ gas first and then H_2_, CO_2_ dissociation proceeds, leaving CO and O adsorbates on the surface. When the order of gas introduction is reversed, COOH formation occurs. The reaction events are closely related to the initial process on the Ni surface.

Figure 9 shows the illustration of the reaction pathways depending on the dosage order of H_2_/CO_2_ gases for high-pressure conditions. In the case when CO_2_ gas is introduced first, surface oxidation proceeds and the Ni(111) surface becomes covered with NiO species that remain dominant even after adding H_2_ gas. In contrast, when H_2_ is introduced first, the Ni(111) surface is initially covered with atomic hydrogen that reacts with the subsequent CO_2_ molecules, leading to COOH formation. COOH species at the surface easily dissociate into CO and OH adsorbates that are relevant intermediates of CO_2_ hydrogenation.

**Figure 9:**
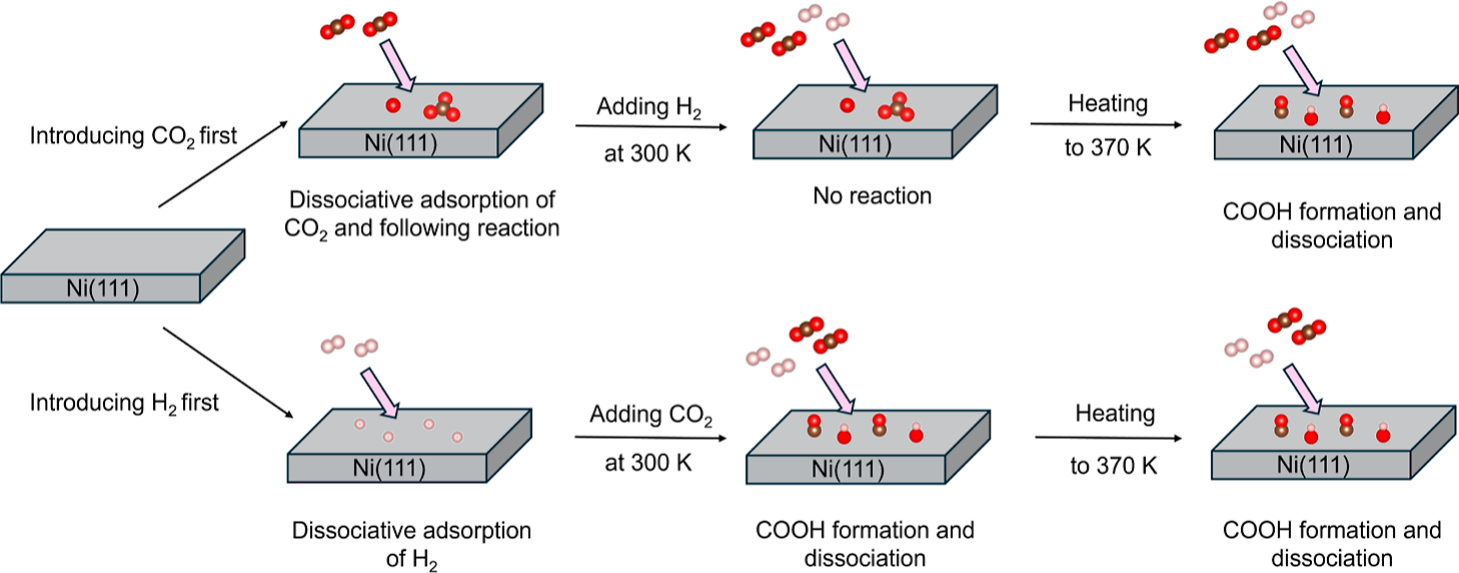
Illustration of the CO_2_/H_2_ reaction pathways on Ni(111) in the case when (above) CO_2_ is introduced first and (below) H_2_ is introduced first for high-pressure conditions.

The surface reaction on Ni(111) has been argued to be complicated owing to observations of various compound species after similar experimental procedures.^9–11^ These previous results can consistently be described by the initial process at the surface, which can be controlled by the dosing order. It is of note that the reaction process is independent of such order at temperatures (>470 K) that are adopted in industrial applications. This fact is seemingly consistent with the conventional consensus of the requirement for high temperatures (typically above 470 K) for the Sabatier reaction because it does not depend on individual operations. Nowadays, catalytic reactions, including the Sabatier process, have been examined to decrease the activation temperature with the aim of reducing energy consumption.

Academic researchers have tackled this research topic based on their understanding of the ideal or well-defined model systems. In this study, we found an actual relationship between the dosage order and the reaction pathway on the Ni(111) surface (Figure 9). The cause was found to be surface chemical initiation. When we expose the Ni(111) surface to the mixed CO_2_/H_2_ gas, the surface reaction at a low temperature most likely occurs first with the H_2_ gas rather than with the CO_2_ gas, implying that the system is associated with the reduced surface of Ni(111). In considering the efficient usages of mixed gas for the Ni-based catalysts, one of the composing gases can be utilized to control the initial surface treatment to promote the reaction. It should be noted that the influences of surface contaminants cannot be completely ruled out in the present experiment. Although further investigation is required to elucidate the Ni surface chemistry, we have shown how the initial pathway of the reaction is selected on Ni(111) on the actual surfaces in the experimental chambers. The surface chemistry still leaves room to rationally design the roles of gases and surface species that will pave the way for developing much more efficient catalysts.

## Conclusion

In summary, we conducted APXPS experiments on Ni(111) under a H_2_/CO_2_ mixed gas atmosphere and observed two types of reaction processes at the surface, depending on the order of gas dosage. CO_2_ dissociation proceeds when CO_2_ gas is introduced first; only carboxyl (COOH) formation occurs when the first gas is H_2_. The difference in the reaction pathways originates from the surface conditions due to the interaction between the initial gas and Ni(111). Under the low-pressure condition (0.4 mbar in total), CO_3_ and NiO formed by CO_2_ dissociation remain on the surface until 370 K and hinder the COOH formation. On the other hand, under the high-pressure condition (10 mbar in total), the reaction is dominated by COOH formation and subsequent dissociation at 370 K and is associated with graphitization above 470 K in both cases. These observations reveal the relationship between the initial catalytic surface and the reaction pathways at annealing temperature, paving a new understanding of achieving CO_2_ hydrogenation catalysts with a lower energy consumption.

## Supplementary Material



## Notes

Series of C 1*s*, O 1*s*, and Ni 2*p*_3/2_ XPS spectra of Ni(111) during heating without gases and the corresponding changes in amounts of carbon species as a function of sample temperature (PDF)
