# Supplementary material for: Selecting Reaction Pathways of CO2 Hydrogenation on Ni(111) by Kinetic Hindrance Associated with the Initial Surface Conditions in the Treatment of H2/CO2 Mixed Gas, Studied by Ambient-Pressure X‑ray Photoelectron Spectroscopy
Source: ACS Omega. 2025 Nov 21;10(48):58238–49. doi: 10.1021/acsomega.5c04196 (PMC12771453; doi:10.1021/acsomega.5c04196)
Supplement: Supplementary file 1 [file ao5c04196_si_001.pdf]

Selecting Reaction Pathways of CO_2 hydrogenation on Ni(111) by Kinetic Hindrance Associated with the Initial Surface Conditions in the Treatment of H_2/CO_2 Mixed Gas, Studied by Ambient-Pressure X-Ray Photoelectron Spectroscopy

Yu Murano,[†] Masafumi Horio,[†] Tetsuya Wada,[†] Masashige Miyamoto,[†] Yifu Liu,[†] Yoshinori Kotani,[‡] Hiroyuki Yamane,[‡] Tetsuya Nakamura,[‡,¶] Susumu Yamamoto,[¶,§] and Iwao Matsuda[*,‖]

†*The Institute for Solid State Physics (ISSP), The University of Tokyo, Kashiwa, Chiba 277-8581, Japan*

‡*Photon Science Innovation Center (PhoSIC), Sendai, Miyagi 980-0845, Japan*

¶*International Center for Synchrotron Radiation Innovation Smart (SRIS), Tohoku University, Sendai, Miyagi 980-8577, Japan*

§*Institute of Multidisciplinary Research for Advanced Materials (IMRAM), Tohoku University, Sendai, Miyagi 980-8577, Japan*

‖*Institute for Solid State Physics (ISSP), The University of Tokyo, Kashiwa, Chiba 277-8581, Japan*

E-mail: imatsuda@issp.u-tokyo.ac.jp

1. XPS measurements during the heating without gases

Figure S1 shows a series of (a) C 1*s*, (b) O 1*s*, and (c) Ni $2p_{3/2}$ spectra during heating the Ni(111) sample from 300 to 570 K under UHV conditions taken at KEK-PF BL-13B. The spectral assignments and curve-fittings follow those of Fig. 2. The changes in carbon to nickel (C/Ni) atomic ratio with temperature are shown in Fig. S2.

The carbon species including graphitic carbons and NiC were observed during the heating (Fig. S1 (a)). Nickel was kept metallic throughout the heating, and almost no oxygen species were detected (Fig. S1 (b) and (c)). Comparing Fig. S2 with Fig. 4 and 7, the carbon species segregate as NiC, while the amounts of graphitic carbons are much lower than those under the reactant gases especially at high temperatures. This indicates that the evolution of such carbon species under CO_2/H_2 comes from the atomic carbons formed by the interaction between the gases and the sample surface.

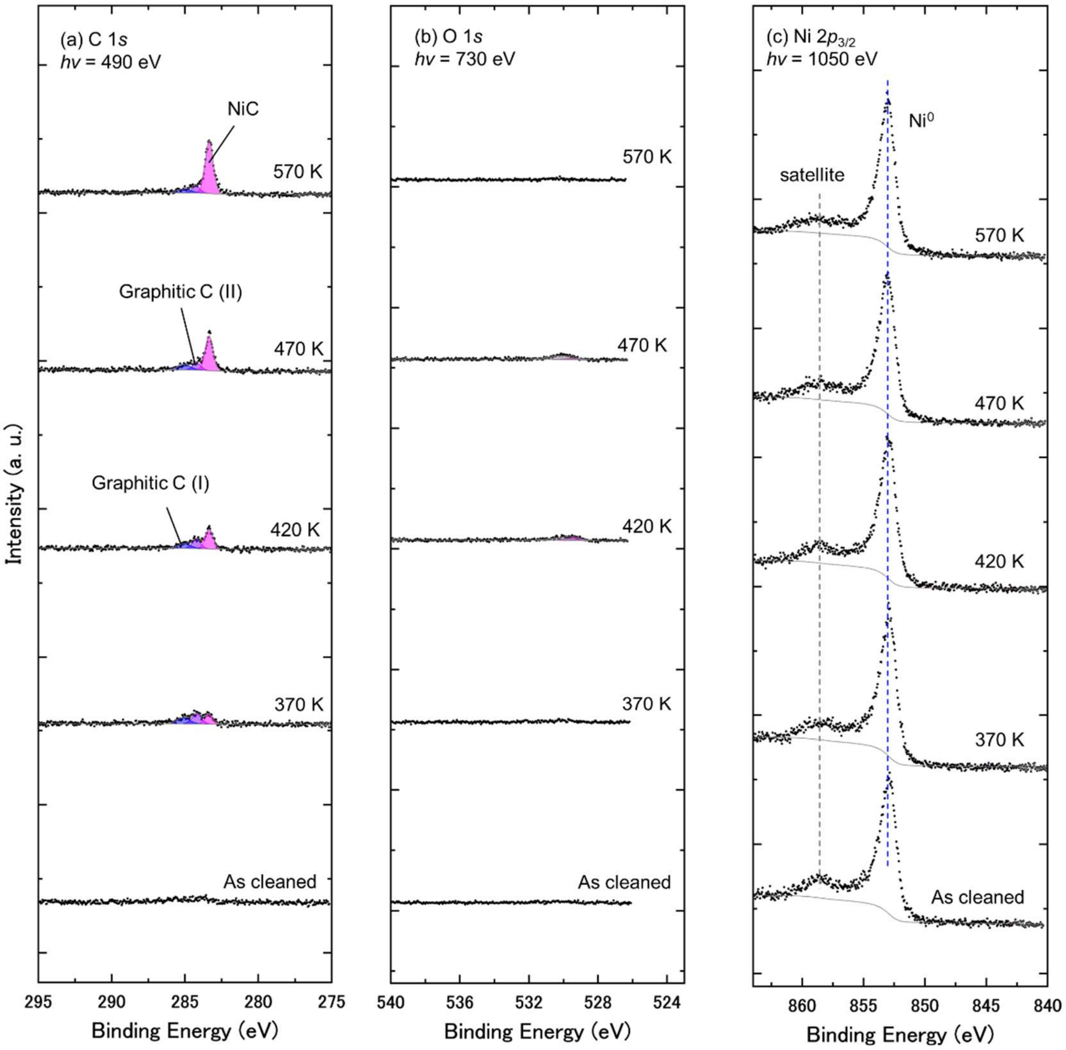

Figure S1. A series of XPS spectra during the heating of a Ni(111) sample from 300 to 570 K under UHV conditions taken at core-levels of (a) C 1*s*, (b) O 1*s*, and (c) Ni $2p_{3/2}$. The C 1*s* and O 1*s* spectra are normalized to the same scale as Fig. 2.

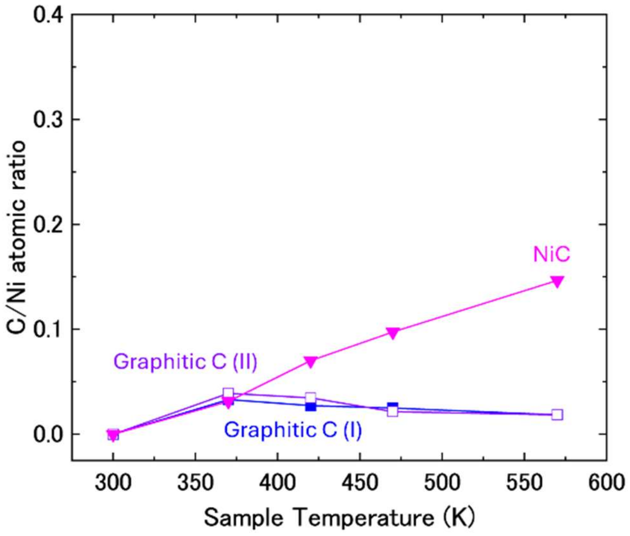

Figure S2. The C/Ni atomic ratio for C 1s species on Ni(111) during heating the sample from 300 to 570 K under UHV conditions.
